# Diffusion-Weighted Imaging, MR Angiography, and Baseline Data in a Systematic Multicenter Analysis of 3,301 MRI Scans of Ischemic Stroke Patients—Neuroradiological Review Within the MRI-GENIE Study

**DOI:** 10.3389/fneur.2020.00577

**Published:** 2020-06-25

**Authors:** Mattias Drake, Petrea Frid, Björn M. Hansen, Ona Wu, Anne-Katrin Giese, Markus D. Schirmer, Kathleen Donahue, Lisa Cloonan, Robert E. Irie, Mark J. R. J. Bouts, Elissa C. McIntosh, Steven J. T. Mocking, Adrian V. Dalca, Ramesh Sridharan, Huichun Xu, Eva Giralt-Steinhauer, Lukas Holmegaard, Katarina Jood, Jaume Roquer, John W. Cole, Patrick F. McArdle, Joseph P. Broderick, Jordi Jiménez-Conde, Christina Jern, Brett M. Kissela, Dawn O. Kleindorfer, Robin Lemmens, James F. Meschia, Tatjana Rundek, Ralph L. Sacco, Reinhold Schmidt, Pankaj Sharma, Agnieszka Slowik, Vincent Thijs, Daniel Woo, Bradford B. Worrall, Steven J. Kittner, Braxton D. Mitchell, Jonathan Rosand, Polina Golland, Arne Lindgren, Natalia S. Rost, Johan Wassélius

**Affiliations:** ^1^Department of Clinical Sciences Lund, Radiology, Lund University, Lund, Sweden; ^2^Department of Radiology, Neuroradiology, Skåne University Hospital, Lund, Sweden; ^3^Department of Clinical Sciences Lund, Neurology, Lund University, Lund, Sweden; ^4^Department of Neurology and Rehabilitation Medicine, Neurology, Skåne University Hospital, Malmö, Sweden; ^5^Department of Radiology, Athinoula A. Martinos Center for Biomedical Imaging, Massachusetts General Hospital (MGH), Harvard Medical School, Charlestown, MA, United States; ^6^Department of Neurology, Massachusetts General Hospital, Harvard Medical School, Boston, MA, United States; ^7^Program in Medical and Population Genetics, Broad Institute of MIT and Harvard, Cambridge, MA, United States; ^8^Department of Population Health Sciences, German Centre for Neurodegenerative Diseases (DZNE), Bonn, Germany; ^9^Computer Science and Artificial Intelligence Laboratory, MIT, Cambridge, MA, United States; ^10^Division of Endocrinology, Diabetes and Nutrition, Department of Medicine, University of Maryland School of Medicine, Baltimore, MD, United States; ^11^Department of Neurology, Neurovascular Research Group (NEUVAS), IMIM-Hospital del Mar (Institut Hospital del Mar d'Investigacions Mèdiques), University at Autonoma de Barcelona, Barcelona, Spain; ^12^Institute of Neuroscience and Physiology, The Sahlgrenska Academy at University of Gothenburg, Gothenburg, Sweden; ^13^Department of Neurology, University of Maryland School of Medicine and Veterans Affairs Maryland Health Care System, Baltimore, MD, United States; ^14^Department of Neurology and Rehabilitation Medicine, University of Cincinnati College of Medicine, Cincinnati, OH, United States; ^15^Department of Laboratory Medicine, Institute of Biomedicine, The Sahlgrenska Academy at University of Gothenburg, Gothenburg, Sweden; ^16^Department of Neurosciences, Experimental Neurology and Leuven Research Institute for Neuroscience and Disease (LIND), Leuven, Belgium; ^17^Department of Neurology, VIB, Vesalius Research Center, Laboratory of Neurobiology, University Hospitals Leuven, Leuven, Belgium; ^18^Department of Neurology, Mayo Clinic, Jacksonville, FL, United States; ^19^Department of Neurology and the Evelyn F. McKnight Brain Institute, Miller School of Medicine, University of Miami, Miami, FL, United States; ^20^Clinical Division of Neurogeriatrics, Department of Neurology, Medical University Graz, Graz, Austria; ^21^Institute of Cardiovascular Research, Royal Holloway University of London (ICR2UL), Egham, United Kingdom; ^22^Ashford and St Peter's Hospital, Surrey, United Kingdom; ^23^Department of Neurology, Jagiellonian University Medical College, Krakow, Poland; ^24^Stroke Division, Florey Institute of Neuroscience and Mental Health, Heidelberg, VIC, Australia; ^25^Department of Neurology, Austin Health, Heidelberg, VIC, Australia; ^26^Departments of Neurology and Public Health Sciences, University of Virginia, Charlottesville, VA, United States; ^27^Geriatric Research and Education Clinical Center, Veterans Administration Medical Center, Baltimore, MD, United States; ^28^Henry and Allison McCancer Center for Brain Health and Center for Genomic Medicine, Massachusetts General Hospital, Boston, MA, United States; ^29^Department of Clinical Sciences Lund, Neurology, Lund University, Lund, Sweden; ^30^Department of Neurology and Rehabilitation Medicine, Neurology, Skåne University Hospital, Lund, Sweden

**Keywords:** stroke, imaging, MRI, phenotype, DWI

## Abstract

**Background:** Magnetic resonance imaging (MRI) serves as a cornerstone in defining stroke phenotype and etiological subtype through examination of ischemic stroke lesion appearance and is therefore an essential tool in linking genetic traits and stroke. Building on baseline MRI examinations from the centralized and structured radiological assessments of ischemic stroke patients in the Stroke Genetics Network, the results of the MRI-Genetics Interface Exploration (MRI-GENIE) study are described in this work.

**Methods:** The MRI-GENIE study included patients with symptoms caused by ischemic stroke (*N* = 3,301) from 12 international centers. We established and used a structured reporting protocol for all assessments. Two neuroradiologists, using a blinded evaluation protocol, independently reviewed the baseline diffusion-weighted images (DWIs) and magnetic resonance angiography images to determine acute lesion and vascular occlusion characteristics.

**Results:** In this systematic multicenter radiological analysis of clinical MRI from 3,301 acute ischemic stroke patients according to a structured prespecified protocol, we identified that anterior circulation infarcts were most prevalent (67.4%), that infarcts in the middle cerebral artery (MCA) territory were the most common, and that the majority of large artery occlusions 0 to 48 h from ictus were in the MCA territory. Multiple acute lesions in one or several vascular territories were common (11%). Of 2,238 patients with unilateral DWI lesions, 52.6% had left-sided infarct lateralization (*P* = 0.013 for χ^2^ test).

**Conclusions:** This large-scale analysis of a multicenter MRI-based cohort of AIS patients presents a unique imaging framework facilitating the relationship between imaging and genetics for advancing the knowledge of genetic traits linked to ischemic stroke.

## Background

Neuroimaging analysis is the cornerstone of modern stroke management, with computed tomography (CT) and magnetic resonance imaging (MRI) being the main modalities (1). Compared to CT, MRI has greater sensitivity and versatility and provides an earlier and more precise method for delineating acute ischemic lesions (2) and also a more detailed assessment of chronic small vessel disease.

Magnetic resonance imaging differentiates acute ischemic lesions from subacute or chronic infarcts better than CT (2, 3). Magnetic resonance imaging can aid in the early etiological assessment of acute ischemic stroke (AIS), by differentiating among hypoperfusion injury, lacunar infarction, and cortical vessel occlusion (4). The addition of magnetic resonance angiography (MRA) further assists in the characterization of the cerebrovascular phenotype (5). These properties have made MRI a common tool in the acute management of ischemic stroke to guide acute intervention, optimize secondary prevention, and improve outcomes prediction.

Several population-based MRI initiatives, such as the Rotterdam and Framingham studies, have provided valuable insights into the prevalence of cerebral infarcts (6, 7), including the distribution with regard to laterality (8, 9). However, there are few large MRI-based stroke registries (10), and additional insights can be gained in understanding the AIS characteristics on MRI across a multiethnic, hospital-based cohort.

Building on the extensive data obtained from clinical stroke patients within the National Institute of Neurological Disorders and Stroke–funded Stroke Genetics Network (SiGN), the MRI-Genetics Interface Exploration (MRI-GENIE) study offers such an opportunity by providing clinical MRIs from 12 hospital-based cohorts of AIS patients enrolled internationally. A detailed description of the design and rationale for both these studies has been published previously (11, 12). The MRI-GENIE initiative has already resulted in a number of pioneering studies in automated volumetric MRI analysis (13–15), posterior-circulation stroke (16), and several ongoing projects using the imaging data to link genetic traits, such as the recently discovered RABEP2-gene (17) with phenotypic outcome.

We capitalized on the robust MRI-GENIE database of 3,301 ischemic stroke cases with genome-wide genotyping and MRI scans by systematically reviewing and characterizing the multimodal clinical scans for specific vascular anatomy and pathology, ischemic lesion location, lateralization, multiplicity, and other characteristics according to a predefined structured reporting protocol. In this article, we report the initial findings from the structured neuroradiological analysis of MRI scans within MRI-GENIE.

## Methods

**Figure 1:**
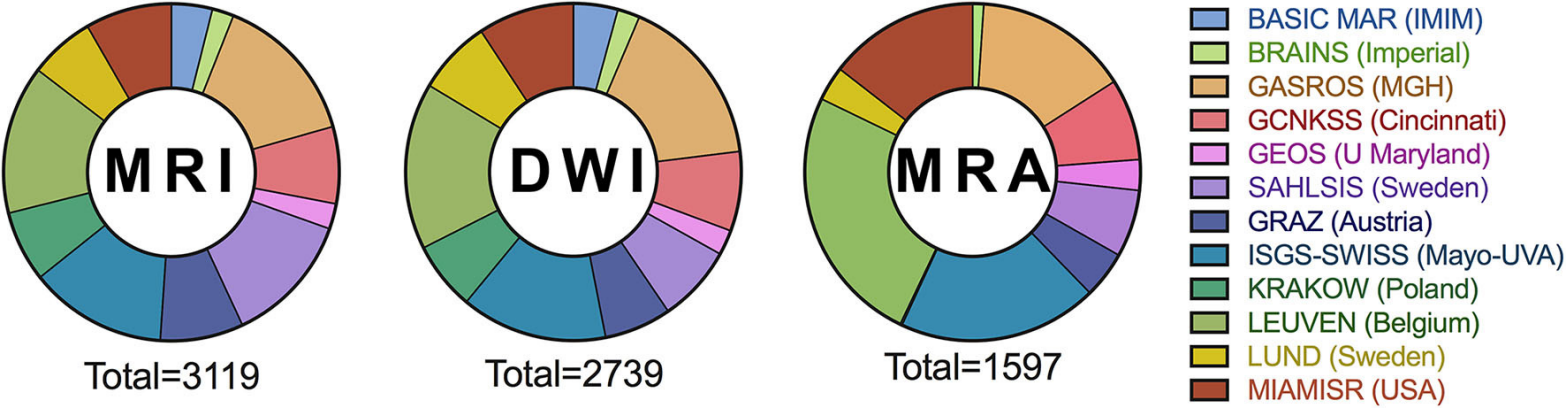
Number of MRI, DWI, and MRA in the MRI-Genie image depository by the contributing center.

MRI-GENIE is an international multicenter collaborative study of ischemic stroke cases for whom clinical MRI scans were obtained on admission for AIS, in addition to genome-wide genotyping subsequently obtained. It is a major international initiative to explore the genetic architecture of MRI traits in AIS patients. As MRI-GENIE was based on the SiGN collaboration, participating sites within SiGN were invited to participate. Participating centers have committed to contribute all available MRI for the patients included within the SiGN collaboration. To date, 12 of the 24 centers have contributed data to the project (Figure 1 and Supplemental Table 1). A Scientific Steering Committee oversees the MRI-GENIE study and critically reviews project proposals facilitating collaborative efforts.

### Patients

All participants provided signed informed consent. Contributed data included demographic and stroke risk factor data, availability of genome-wide genotyping data, and clinical MRI scan. Stroke subtypes were classified in the parent SiGN Study according to the Causative Classification System for Ischemic Stroke (CCS), and Trial of Org 10 172 in Acute Stroke Treatment (TOAST) classification systems (18).

We deidentified the clinical MRI examinations from contributing centers, uploaded the DICOM format images in the MRI-GENIE image repository, and systematically annotated the imaging sequences (12).

### Radiological Characteristics

Two licensed neuroradiologists (J.W. and M.D.) reviewed all MRI examinations *via* the web interface (12). They remained blinded to clinical information, stroke phenotype data, and any diagnostic information from the contributing center to avoid bias of image interpretation. A structured reporting protocol was implemented in close collaboration between the coinvestigators and utilized for all reviewed cases. Interrater agreement was measured in a portion of cases (446 cases, 13.5%) reviewed by both assessors.

The protocol included assigning the ischemic lesions observed on the diffusion-weighted images (DWIs) to predefined anatomical categories, arterial supply regions, territories of the major cerebral arterial branches, and angiographic data to categories for predefined vascular segments. When assigning lesions to the predefined anatomical categories basal and vascular segments, a generally accepted map of the major cerebral vascular territories was used (Supplemental Figure 1). Ischemic lesions from DWI were further assigned as cortical and/or subcortical, single/multiple, and—for the supratentorial subcortical lesions—lacunar or nonlacunar. A single subcortical, supratentorial lesion smaller than 1.5 cm was further defined as lacunar. Volumetric analysis of the DWIs was recently published (14).

The reviewers determined vessel stenosis (stenosis visually gauged to >50%) and occlusion on MRA images. The reviewers also determined the proportion of patients with large artery occlusion (LAO) based on information on occlusion and lesion location on the DWI sequences.

Unexpected findings such as tumors and aneurysms were noted, but not categorized further. The structured reporting protocol is detailed in Supplemental Table 2.

### Statistics

Data were processed using SPSS Statistics (version 24; IBM Corporation, Armonk, NY, USA). Descriptive statistics, including means with standard deviations (SDs) and medians with interquartile range (IQR), were reported where applicable. The χ^2^ test was used to compare proportions of left- vs. right-sided cerebral infarcts among all patients, as well as patients with anterior circulation infarcts only. Patients with bilateral infarcts or no visible infarct were excluded. Level of significance was set to *P* < 0.05.

## Results

### Study Population

Of the 3,301 correctly included patients in the image repository, 183 were unavailable for analysis due to technical errors. Additionally, 18 patients in the image repository had not been correctly included and were therefore excluded from the analysis.

**Figure 2:**
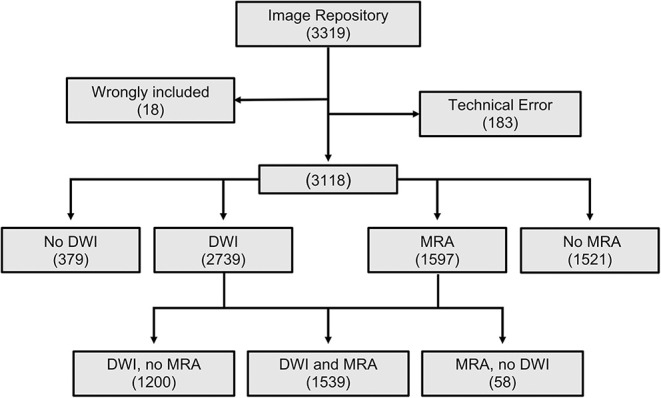
CONSORT diagram of the MRI-GENIE study population.

A flowchart describing the material, including available MRI sequences, is shown in Figure 2. Diffusion-weighted image was available for 2,739 patients (median age = 65 years, IQR = 53–75 years; 39.4% female), whereas MRA was available for 1,597 patients (median age = 64 years, IQR = 52–75 years; 39.3% female). Both DWI and MRA were available for 1,539 patients (median age = 64 years, IQR = 52–75 years; 39.4% female). Technical specifications such as MRI manufacturer, model names, and field strengths are shown in the Supplemental Table 3.

The median time from symptom onset to MRI for the 2,739 patients with available DWI was 1 day (IQR = 1–4 days). In addition, 1,227, 1,061, and 123 of these patients had an MRI performed in the time intervals 0 to 48 h, 2 to 14 days, and more than 14 days, respectively, as shown in Table 1.

**Table 1:** Diffusion-weighted image lesion characteristics related to time from stroke to MRI (328 patients lacked information about time to DWI).

**DWI parameter**	**0–48 h**	**2–14 d**	**>14 d**
Number of patients	1,227	1,061	123
Median time to MRI (days) (IQR)	1 (0–1)	3 (2–6)	27 (20–37)
Anterior/posterior circulation/both (*N*)	762/324/39	676/278/31	64/25/1
Supratentorial/infratentorial/both (*N*)	886/179/63	759/176/50	71/16/3
Right/Left/both (*N*)	486/539/104	434/464/86	40/45/5
Brainstem, *N* (%)	90 (7.3)	103 (9.8)	8 (6.5)
Cerebellum, *N* (%)	62 (5.1)	59 (5.6)	6 (4.9)
Basal ganglia (%)	189	157	10
Hemisphere (%)	658	563	60
Infarct not seen, *N* (%)	99 (8.1)	76 (7.2)	33 (26.8)
Multiple infarcts, *N* (%)	129 (10.5)	103 (9.7)	6 (4.9)

### Center Differences

The 12 participating centers contributed 70–475 patients. Center-specific demographics and radiological data are included in Supplemental Table 1.

### Stroke Etiology According to CCS and TOAST

All 3,301 correctly included subjects in the image repository had a stroke etiology classification according to CCS, and 89.6% had a TOAST assessment (Supplemental Tables 1A–D).

### Risk Factor Distribution

Hypertension was the most common cardiovascular risk factor, present among 65% of the 3,301 cases (0.7% missing data). Additionally, 51% were current or former smokers (0% missing), 23% had diabetes mellitus (0.9% missing), 18% had coronary artery disease (2.3% missing), and 14% had atrial fibrillation (1.1% missing).

### Radiological Characteristics

#### DWI Lesion Characteristics

**Figure 3:**
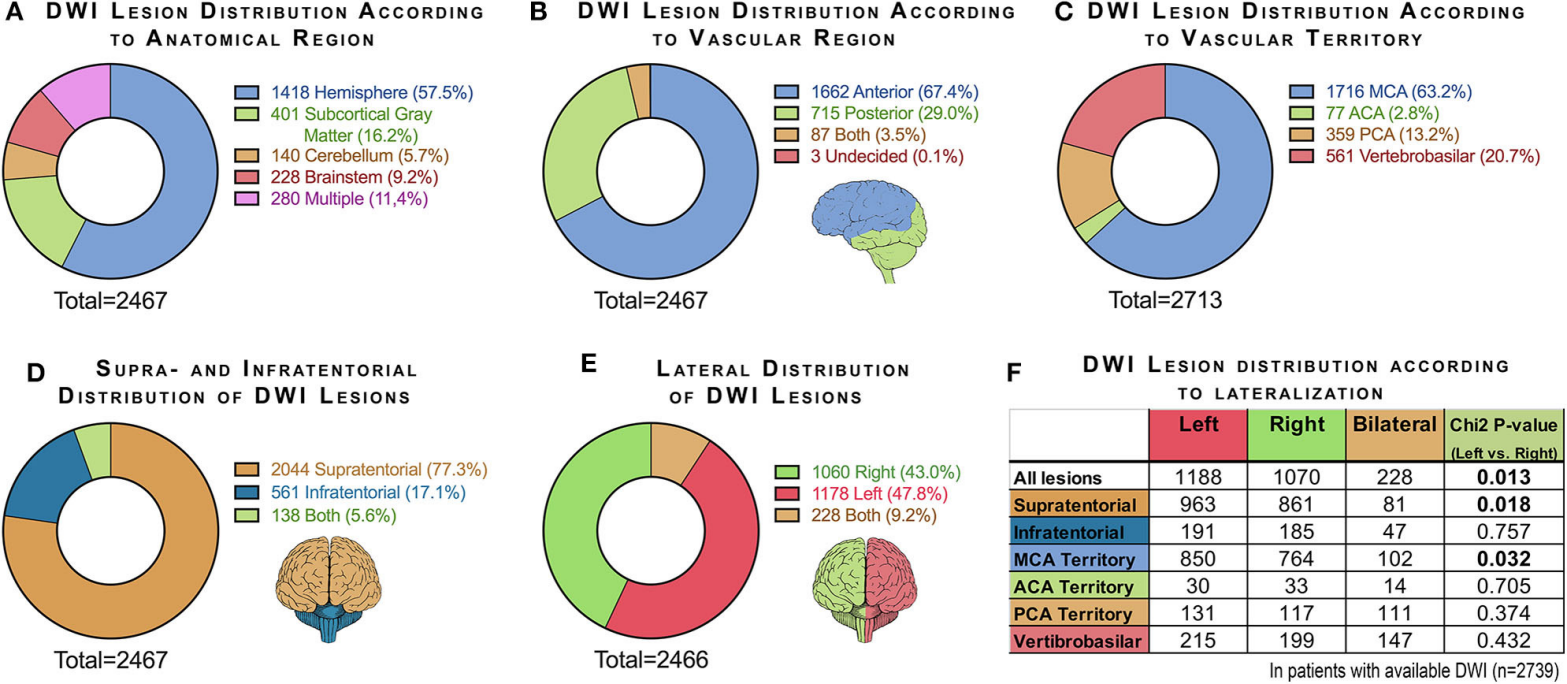
Charts summarizing DWI lesion distribution according to **(A)** anatomical region, **(B)** vascular region, **(C)** vascular territory, **(D)** supratentorial and infratentorial location, and **(E)** lateralization. In chart **(C)**, multiple infarcts were recorded once per vascular territory, and therefore the number of infarcts (2,713) exceeds the total number of patients with visible DWI lesions (2,467). Chart **(F)** shows the difference between right- and left-sided lesions for all lesions and specified for supratentorial and infratentorial lesions and specified for the major vascular territories [χ^2^ test after excluding patients with bilateral infarcts and with no infarcts (270 cases)].

Ischemic lesions on the available DWI (*n* = 2,739) were assigned to predefined anatomical categories based on normal cerebral anatomy and vascular territories (Supplemental Figure 1) with further classification into solitary or multiple lesions. Ischemic lesion distributions are described in detail in Figure 3. Interrater agreement assessed in the 443 cases independently reviewed by both assessors was 98%, with Cohen unweighted κ = 0.96 (excellent) and weighted κ = 0.97 (excellent) measured using a single review criterion (vascular territory).

The most common anatomical category based on DWI was hemispheric (51.7%). The second most common ischemic lesion location was deep gray matter (defined as basal ganglia and thalamus), seen in 14.6% of the examinations.

In 2,044 (77%) of the examinations with lesions on DWIs, supratentorial lesions were seen; in 561 (17%), infratentorial lesions were seen, and in 138 examinations (6%), both supratentorial and infratentorial lesions were seen.

Overall, 67% of the ischemic lesions occurred in the anterior circulation, 29% in the posterior circulation, 3.5% to both anterior and posterior circulation, and 0.1% undetermined. When further stratified by vascular territory according to the major arterial branches, the middle cerebral artery (MCA) territory was most common, followed by vertebrobasilar artery territory and posterior cerebral artery territory.

Of the 2,238 patients with unilateral infarcts seen on DWI, 53% had left-sided infarct lateralization (*P* = 0.013, Figure 3F). When comparing the distribution of unilateral infarctions for supratentorial and infratentorial distribution, a higher frequency of left-sided lesions was seen only for supratentorial infarcts (53%; *P* = 0.018). When comparing the distribution of unilateral infarctions for the major vascular territories, a significantly higher frequency of left-sided infarcts was seen only for the MCA territory (53%; *P* = 0.032).

#### MRA Characteristics of Intracranial Vessels

Among the 1,597 patients with available MRA of the intracranial vessels, moderate to severe vessel stenosis (stenosis visually gauged to >50% luminal diameter reduction) was most common in the basilar artery and the vertebral arteries (4% and 3%, respectively). Stenosis was observed in 1% to 2% of the large vessels of the anterior circulation and the posterior cerebral artery. Vessel occlusion was most common in the MCA (14%) and the vertebral arteries (12%), whereas occlusion of the basilar artery was least common (2%).

**Figure 4:**
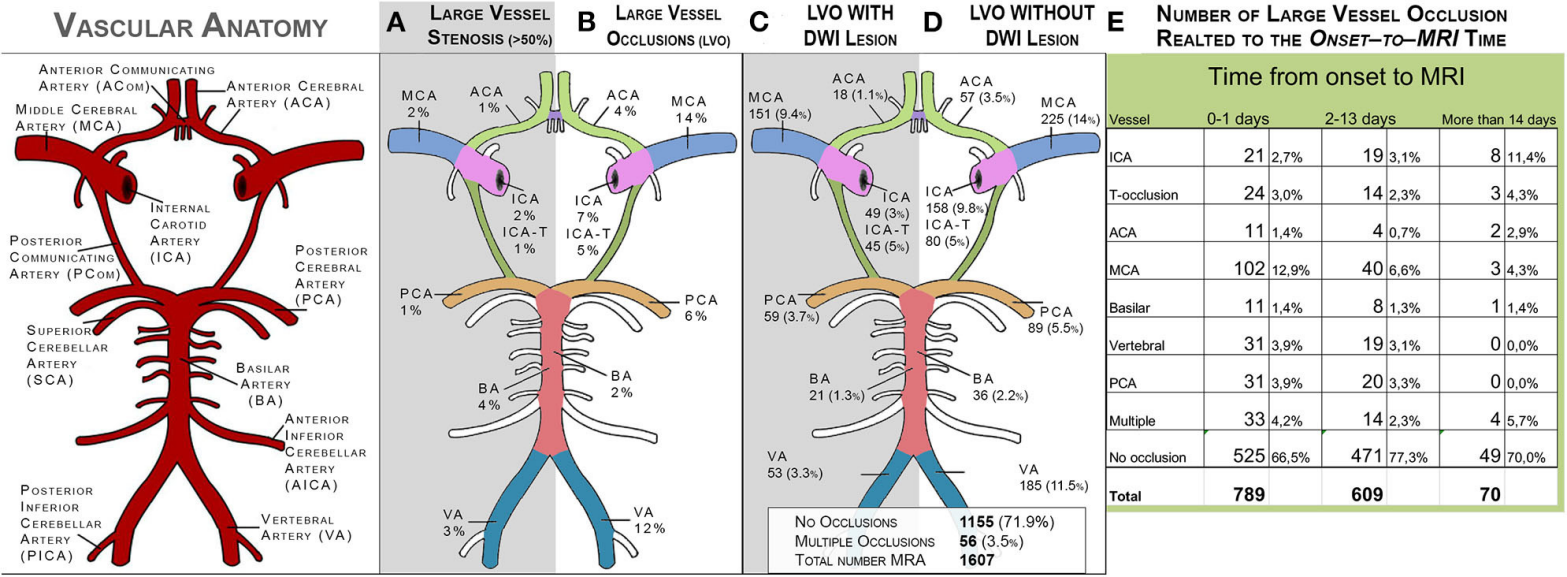
Distribution of stenosis and occlusions in the large intracranial vessels on MRA (*N* = 1,597). **(A)** shows the distribution of stenosis (visually graded as >50%). **(B)** shows the distribution of large-vessel occlusions. **(C)** shows the distribution of large-vessel occlusions with corresponding DWI lesions. **(D)** shows the distribution of large-vessel occlusions without corresponding DWI lesions. ACA, anterior cerebral artery; MCA, middle cerebral artery; ICA, internal carotid artery; ICA-T, top of internal carotid artery; PCA, posterior cerebral artery; BA, basilar artery; VA, vertebral artery.

Figure 4 illustrates the distribution of stenosis in the major intracranial arterial branches and LAOs with or without corresponding DWI lesions, as well as the distribution of LAOs in relation to the time from stroke onset to MRI. Of the 1,539 patients with available DWI and MRA, 423 (28%) had an large vessel occlusion (LVO) relevant to the localization of an infarct on DWI (Table 2). The proportion of patients with LAO was higher in MRAs performed within 48 h of stroke onset (37%) compared with MRAs performed later than 48 h (23%) after stroke. Middle cerebral artery occlusions accounted for more than a third (34%) of all LAOs, whereas basilar artery occlusion and anterior cerebral artery occlusion were the rarest occlusion types (4.7% and 4.2% of all LAOs, respectively).

**Table 2:** Large artery occlusions (LAO) in the 1,507 patients with DWI and MRA (32 patients lacked information about time to DWI).

**Arterial segment**	**0–48 h (*n* = 803)**	**2–14 days (*n* = 635)**	**>14 days (*n* = 69)**
Proximal internal carotid, *N* (%)	21 (2.6)	20 (3.1)	7 (10.1)
Distal internal carotid, *N* (%)	25 (3.1)	15 (2.4)	3 (4.3)
Middle cerebral artery, *N* (%)	100 (12.4)	41 (6.5)	2 (2.9)
Anterior cerebral artery, *N* (%)	11 (1.4)	5 (0.8)	2 (2.9)
Vertebral artery, *N* (%)	31 (3.9)	19 (3.0)	0
Basilar artery, *N* (%)	11 (1.4)	8 (1.3)	1 (1.4)
Posterior cerebral artery, *N* (%)	31 (3.9)	20 (3.1)	0
Multiple arteries, *N* (%)	32 (4.0)	17 (2.7)	1 (1.4)
No occlusion visible, *N* (%)	541 (67.3)	490 (77.2)	53 (76.8)

## Discussion

In this systematic radiological analysis of clinical MRI scans of 3,301 AIS patients enrolled in 12 international hospital-based cohorts, we identified that anterior circulation infarcts were most prevalent (67%), that infarcts in the MCA territory were the most common, and that the majority of LAOs 0 to 48 h from ictus were in the MCA territory. It was also observed that the proportion of large-vessel occlusions decreased with increasing time from onset to imaging, especially for the MCA territory.

Multiple acute lesions in one or several vascular territories were common (11%), and this prevalence was comparable with other current studies (8.8–9.8%) (19). However, the two major etiologies for multiple acute DWI lesions, large artery atherosclerosis and cardioembolic, were notably common in the cohort (36 and 40%, CCS and TOAST), suggesting that most patients with stroke attributed to cardioembolic and large-artery atherosclerosis present with a single DWI lesion.

We also showed a higher prevalence of left-sided ischemic lesions compared to right-sided lesions and that this difference in prevalence also could be observed for the largest subgroups of patients with supratentorial lesions and lesions in the MCA territory. Left-sided lesions were also more common on CT in a recent population-based cohort (8, 9). In our material, this difference is caused mainly by a difference in supratentorial lesions in the MCA territory.

These results highlight how “big data” allow for stratification of patients into smaller but more homogeneous subgroups based on lesion appearance on MRI, an important step to individualizing stroke care. Our structured data on imaging and clinical characteristics provide baseline information to assess the feasibility of studies on specific MRI phenotypes such as patients with multiple lesions or lesions in a certain vascular territory. Additionally, our data can be used in the further development and validation of automated segmentation software by providing a very large annotated data set based on standardized neuroradiological assessment.

This unique database also provides an analytical platform for novel, large-scale studies of genetic architecture of MRI-based phenotypes in patients with AIS. To date, there are limited data on the genetics of imaging phenotypes, such as white matter hyperintensities (WMH) and DWI volumes (13, 14). Furthermore, there are limited large-scale studies of genetic architecture and MRI-based phenotypes from hospital-based multicenter stroke populations (20). These data are critical to obtain to advance knowledge of underlying mechanisms of ischemic tissue injury and for developing targets for future interventions and therapeutics. MRI-GENIE offers a novel approach to developing such type of data, and systematic review and radiological analysis of the MRI data are the first steps toward detailed characterization of clinical MRI phenotypes. We will continue our work by combining this work with recently developed automated volume segmentation algorithms (14) to assess the combined effect of risk factors and vascular anatomy with lesion characteristics. We will also assess the effect of genetic traits that may alter the MRI stroke phenotype in general, or in specific conditions such as in the presence of certain neurovascular conditions or certain risk factors.

Limitations of the current approach include known and potential differences between the individual contributing sites. Because this is a hospital-based study, the use of MRI may have varied with clinical practice over time and between centers. The low mean patient age at several centers (Supplemental Table 1A) suggests that MRI may predominantly have been used for younger patients. For example, MRI may have been used predominantly for cases where CT had not been conclusive, thereby underrepresenting cases of large infarcts, or for suspected ischemic strokes in the posterior fossa where CT is of limited diagnostic value because of artifacts in this region (21). However, the proportion of posterior circulation lesions in our cohort matches previous estimates (15–35%) (16, 22). Another limitation is that the MRI scanners and examination protocols varied between centers and over time.

The strengths of our approach include a large sample of MRI scans from multiple international centers obtained as part of hospital-based care for patients with AIS, thereby likely generalizable to global stroke patient population as compared to previously published single-center studies. Additional strengths include the large number of DWI and MRAs, as well as detailed stroke subtype characterization using validated TOAST and CCS classifications.

## Conclusions

In this article, we report baseline data from the MRI-GENIE study and the initial neuroradiological findings describing ischemic lesions, vascular anatomy, and vascular lesions according to a structured reporting protocol.

This work will provide an imaging framework for genetic studies of the MRI-GENIE cohort, facilitating the linkage between imaging and genetics in advancing the knowledge of ischemic stroke. It may also serve as a reference material for automated analysis of ischemic lesions or vascular characteristics.

## Data Availability Statement

The raw data supporting the conclusions of this article will be made available by the authors, without undue reservation, to any qualified researcher.

## Ethics Statement

The studies involving human participants were reviewed and approved by All 12 sites in the SIGN collaboration obtained ethics committee approval for its part of the project, as well as signed informed consent from all included patients. The patients/participants provided their written informed consent to participate in this study.

## Author Contributions

All authors listed have made a substantial, direct and intellectual contribution to the work, and approved it for publication.

## Conflict of Interest

OW: Consulting for Penumbra, Inc; Advisory Board Member for Genentech, Inc. JRos: Consulting for Boehringer Ingelheim and New Beta Innovation. AL: Bayer, BMS/Pfizer, Astra Zeneca and Portola, outside the submitted work. JW: Co-founder and shareholder of UmanSense AB, Sweden. The remaining authors declare that the research was conducted in the absence of any commercial or financial relationships that could be construed as a potential conflict of interest.
