# Supplementary material for: Diffusion-Weighted Imaging, MR Angiography, and Baseline Data in a Systematic Multicenter Analysis of 3,301 MRI Scans of Ischemic Stroke Patients—Neuroradiological Review Within the MRI-GENIE Study
Source: Front Neurol. 2020 Jun 25;11:577. doi: 10.3389/fneur.2020.00577 (PMC7330135; doi:10.3389/fneur.2020.00577)
Supplement: Supplementary file 1 [file Data_Sheet_1.docx]

# Supplementary Material

**Supplemental Figure 1**

##
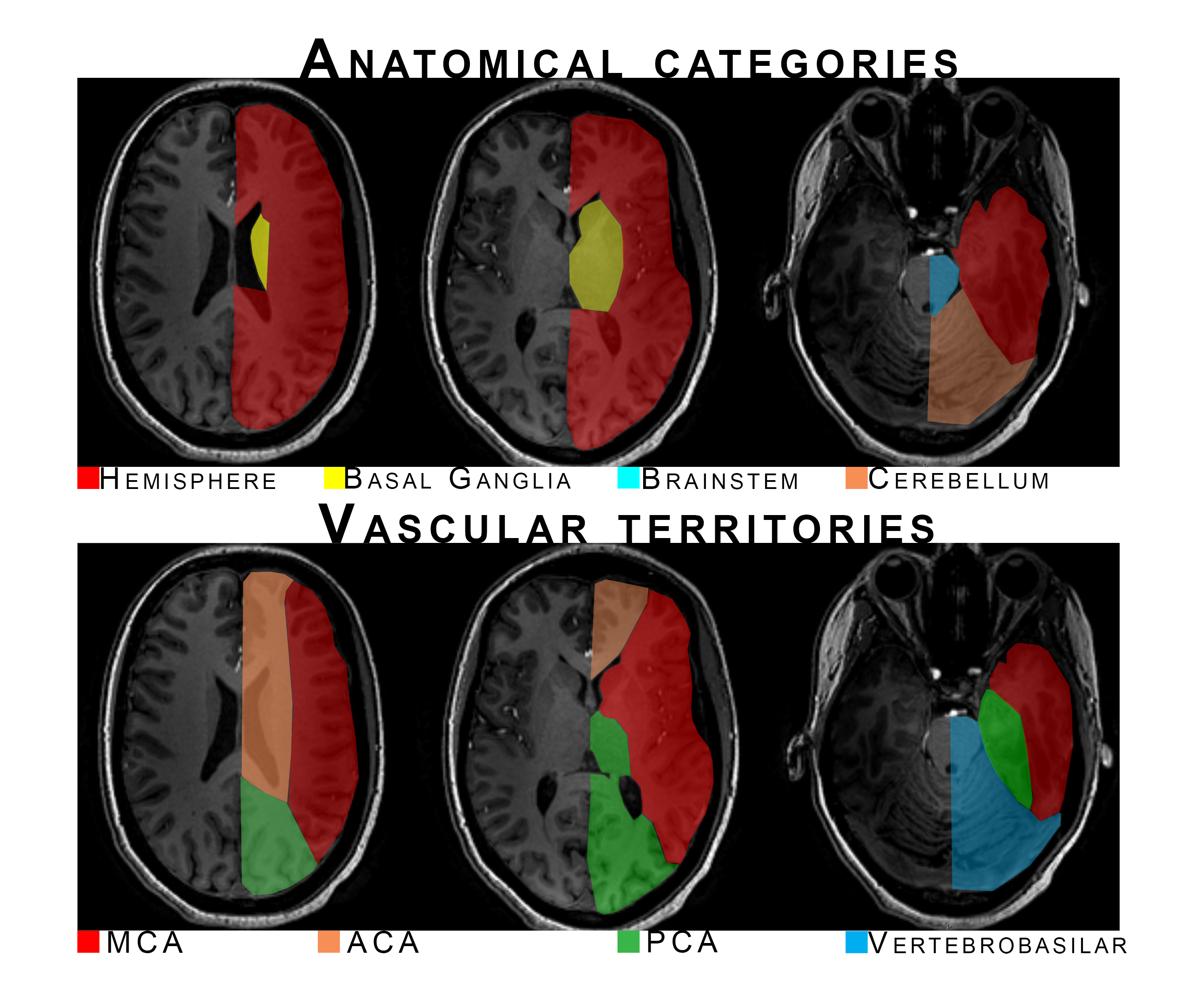


**Supplemental Figure 1**

Schematic illustration of the anatomical categories and vascular territories used when describing ischemic lesions. MCA=Middle Cerebral Artery, ACA=Anterior Cerebral Artery, PCA=Posterior Cerebral Artery.

Combining anatomical categories with vascular territories can be done to identify more specific areas; for example, Thalamic lesions can be identified by combining the subcortical grey matter category with the Posterior Cerebral Artery territory.

## Supplemental Table 1A-D

## Table 1A.

| **Center Name** | **Total (N)** | **Technical error (N)** | **No DWI (N)** | **Infarct not seen (N)** | **Mean age**  **years (SD)** | **Female (%)** | **MRI available (N)** | **DWI available (N)** | **MRA available (N)** | **DWI+MRA (N)** |
| --- | --- | --- | --- | --- | --- | --- | --- | --- | --- | --- |
| BASICM | 124 | 1 | 6 | 7 | 70 (11) | 37 | 123 | 117 | 0 | 0 |
| BRAINS | 70 | 5 | 6 | 24 | 63 (16) | 47 | 65 | 59 | 17 | 17 |
| GASROS | 457 | 2 | 0 | 34 | 65 (14) | 35 | 455 | 455 | 238 | 238 |
| GCNKSS | 245 | 15 | 21 | 19 | 65 (14) | 49 | 230 | 209 | 125 | 118 |
| GEOS | 76 | 0 | 10 | 9 | 42 (7) | 26 | 76 | 66 | 47 | 38 |
| SAHLSIS | 401 | 8 | 191 | 24 | 52 (12) | 39 | 393 | 202 | 103 | 93 |
| GRAZ | 373 | 123 | 73 | 15 | 63 (14) | 30 | 250 | 177 | 72 | 66 |
| ISGS | 425 | 12 | 27 | 37 | 65 (15) | 41 | 413 | 386 | 307 | 291 |
| KRAKOW | 224 | 12 | 32 | 29 | 60 (14) | 45 | 212 | 180 | 3 | 3 |
| LEUVEN | 448 | 4 | 7 | 50 | 67 (15) | 42 | 445 | 438 | 400 | 395 |
| LUND | 196 | 0 | 0 | 17 | 63 (13) | 39 | 196 | 196 | 53 | 53 |
| MIAMIS | 262 | 2 | 6 | 6 | 62 (14) | 37 | 260 | 254 | 232 | 227 |
| **TOTAL** | **3301** | **183** | **379** | **271** | **62 (14)** | **39** | **3118** | **2739** | **1597** | **1539** |

## Table 1B

| **CCS main type** | | | | | | | |
| --- | --- | --- | --- | --- | --- | --- | --- |
| **Center Name** | **Total (N)** | **SAO (%)** | **LAA (%)** | **CE (%)** | **OTHER (%)** | **UNDETERMINED (%)** | **Missing (%)** |
| BASICM | 124 | 37 | 36 | 18 | 0 | 10 | 0 |
| BRAINS | 70 | 7 | 13 | 14 | 27 | 39 | 0 |
| GASROS | 457 | 16 | 20 | 16 | 7 | 41 | 0 |
| GCNKSS | 245 | 29 | 17 | 17 | 2 | 34 | 0 |
| GEOS | 76 | 15 | 21 | 7 | 16 | 42 | 0 |
| SAHLSIS | 401 | 9 | 17 | 6 | 16 | 52 | 0 |
| GRAZ | 373 | 17 | 21 | 20 | 4 | 39 | 0 |
| ISGS | 425 | 9 | 28 | 13 | 7 | 43 | 0 |
| KRAKOW | 224 | 14 | 15 | 11 | 7 | 53 | 0 |
| LEUVEN | 448 | 7 | 21 | 20 | 6 | 45 | 1 |
| LUND | 196 | 26 | 14 | 13 | 9 | 38 | 0 |
| MIAMIS | 262 | 18 | 23 | 18 | 10 | 31 | 0 |
| **TOTAL** | **3301** | **15** | **21** | **15** | **8** | **41** | **0** |

## Table 1C

| **TOAST main type** | | | | | | | |
| --- | --- | --- | --- | --- | --- | --- | --- |
| **Center Name** | **Total (N)** | **SAO (%)** | **LAA (%)** | **CE (%)** | **OTHER (%)** | **UNDETERMINED (%)** | **Missing (%)** |
| BASICM | 124 | 40 | 36 | 24 | 0 | 0 | 0 |
| BRAINS | 70 | 41 | 33 | 11 | 0 | 15 | 23 |
| GASROS | 457 | 11 | 16 | 37 | 4 | 20 | 11 |
| GCNKSS | 245 | 23 | 14 | 18 | 2 | 42 | 0 |
| GEOS | 76 | 16 | 11 | 18 | 8 | 47 | 0 |
| SAHLSIS | 401 | 15 | 16 | 10 | 15 | 45 | 0 |
| GRAZ | 373 | 20 | 22 | 31 | 5 | 23 | 19 |
| ISGS | 425 | 14 | 21 | 23 | 6 | 36 | 0 |
| KRAKOW | 224 | 8 | 13 | 27 | 6 | 46 | 0 |
| LEUVEN | 448 | 13 | 16 | 31 | 5 | 36 | 1 |
| LUND | 196 | 0 | 0 | 0 | 0 | 0 | 100 |
| MIAMIS | 262 | 22 | 25 | 28 | 8 | 16 | 3 |
| **Total** | **3301** | **15** | **17** | **23** | **6** | **29** | **10** |

## Table 1D

| **Risk Factor Prevalence** | | | | | |
| --- | --- | --- | --- | --- | --- |
| **Center Name** | **Hypertension (%)** | **Diabetes Mellitus (%)** | **Atrial Fibrillation (%)** | **Coronary Artery Disease (%)** | **Smoking (Current or former) (%)** |
| BASICM | 73 | 36 | 22 | 11 | 57 |
| BRAINS | 70 | 11 | 13 | 7 | 40 |
| GASROS | 63 | 20 | 15 | 22 | 60 |
| GCNKSS | 77 | 39 | 14 | 34 | 65 |
| GEOS | 33 | 7 | 1 | 4 | 66 |
| SAHLSIS | 54 | 15 | 6 | 9 | 35 |
| GRAZ | 61 | 30 | 21 | 16 | 37 |
| ISGS | 66 | 24 | 14 | 20 | 65 |
| KRAKOW | 66 | 27 | 13 | 19 | 35 |
| LEUVEN | 62 | 18 | 20 | 16 | 51 |
| LUND | 71 | 23 | 9 | 21 | 62 |
| MIAMIS | 79 | 24 | 16 | 13 | 46 |
| Missing | 1 | 1 | 1 | 2 | 3 |
| **Total** | **65** | **23** | **15** | **18** | **51** |

## Supplemental table 1A-D

Clinical and radiological parameters shown for each contributing center. The tables include basic radiological characteristics including availability of DWI and MRA (1A), stroke subtype according to CCS- (1B) and TOAST-classifications (1C), and prevalence of risk factors (1D) reported by the contributing centers. Contributing centers*:* BASICM, IMIM-Hospital del Mar, Barcelona, Spain; BRAINS, Imperial College London, UK; GASROS, Massachusetts General Hospital, Boston, MA, USA; GCNKSS, University of Cincinnati, Cincinnati, OH, USA; GEOS, University of Maryland, Baltimore, MD, USA; SAHLSIS, University of Gothenburg, Sweden; GRAZ, Medical University of Graz, Austria; ISGS, Mayo Clinic Florida, Jacksonville, FL, USA; KRAKOW, Jagiellonian University, Kraków, Poland; LEUVEN, University Hospitals Leuven, Belgium; LUND, Skåne University Hospital, Lund, Sweden; MIAMIS, University of Miami, FL, USA*.*

## Supplemental Table 2

## Supplemental Table 2A

| **Ischemic lesion parameters (categories)** | |
| --- | --- |
| Infarct location according to core lab | Basal ganglia left Basal ganglia right Brainstem left Brainstem right Cerebellum left Cerebellum right Hemisphere left Hemisphere right Multiple Not seen |
| Supratentiorial or infratentorial (None, Supratentorial, Infratentorial, Both) | |
| Lesion lateralization (Left, Right, Both) | |
| Lesion in left brainstem (Yes/No) | |
| Lesion in right brainstem (Yes/No) | |
| Multiple lesions in brainstem (Yes/No) | |
| Only one lesion in brainstem (Yes/No) | |
| Lesion in left cerebellum (Yes/No) | |
| Multiple lesions in left cerebellum (Yes/No) | |
| Only one lesion in left cerebellum (Yes/No) | |
| Lesion in right cerebellum (Yes/No) | |
| Multiple lesions in right cerebellum (Yes/No) | |
| Only one lesion in right cerebellum (Yes/No) | |
| Cortical lesion in left PCA territory (Yes/No) | |
| Subcortical lesion in left PCA territory (Yes/No) | |
| Multiple lesions in left PCA territory (Yes/No) | |
| Only one lesion in left PCA territory (Yes/No) | |
| Cortical lesion in right PCA territory (Yes/No) | |
| Subcortical lesion in right PCA territory (Yes/No) | |
| Multiple lesions in right PCA territory (Yes/No) | |
| Only one lesion in right PCA territory (Yes/No) | |
| Cortical lesion in left MCA territory (Yes/No) | |
| Subcortical lesion in left MCA territory (Yes/No) | |
| Multiple lesions in left MCA territory (Yes/No) | |
| Only one lesion in left MCA territory (Yes/No) | |
| Cortical lesion in right MCA territory (Yes/No) | |
| Subcortical lesion in right MCA territory (Yes/No) | |
| Multiple lesions in right MCA territory (Yes/No) | |
| Only one lesion in right MCA territory (Yes/No) | |
| Cortical lesion in left ACA territory (Yes/No) | |
| Subcortical lesion in left ACA territory (Yes/No) | |
| Multiple lesions in left ACA territory (Yes/No) | |
| Only one lesion in left ACA territory (Yes/No) | |
| Cortical lesion in right ACA territory (Yes/No) | |
| Subcortical lesion in right ACA territory (Yes/No) | |
| Multiple lesions in right ACA territory (Yes/No) | |
| Only one lesion in right ACA territory (Yes/No) | |
| Lacunar lesion (subcortical lesion <1·5 cm on DWI) left PCA territory (Yes/No) | |
| Lacunar lesion (subcortical lesion <1·5 cm on DWI) right PCA territory (Yes/No) | |
| Lacunar lesion (subcortical lesion <1·5 cm on DWI) left MCA territory (Yes/No) | |
| Lacunar lesion (subcortical lesion <1·5 cm on DWI) right MCA territory (Yes/No) | |

## Supplemental Table 2B

| **Vascular parameters (categories)** |
| --- |
| Vascular territory of lesion (Anterior, Posterior, Both, Undecided) |
| Basilar artery status (Open, Stenosis > 50%, Occlusion) |
| Left vertebral status (Open, Stenosis > 50%, Occlusion) |
| Right vertebral status (Open, Stenosis > 50%, Occlusion) |
| Left Pcom status (Not visible, < P1 segment, > P1 segment) |
| Left P1 status (Not visible, < Pcom, > Pcom) |
| Left fetal type PCA i.e. Pcom > P1 segment (Yes/No) |
| Left PCA status (Open, Stenosis > 50%, Occlusion) |
| Right Pcom status (Not visible, < P1 segment, > P1 segment) |
| Right P1 status (Not visible, < Pcom, > Pcom) |
| Right fetal type PCA i.e. Pcom > P1 segment (Yes/No) |
| Right PCA status (Open, Stenosis > 50%, Occlusion) |
| Acom status (Not visible, Open) |
| Left distal ICA status to identify T-occlusion (Open, Stenosis > 50%, Occlusion) |
| Left ACA status (Open, Stenosis > 50%, Occlusion) |
| Left MCA status (Open, Stenosis > 50%, Occlusion) |
| Left ICA status (Open, Stenosis > 50%, Occlusion) |
| Right ACA status (Open, Stenosis > 50%, Occlusion, Open) |
| Right MCA status (Open, Stenosis > 50%, Occlusion, Open) |
| Right ICA status (Open, Stenosis > 50%, Occlusion) |
| Aneurysm detected (Yes/No) |
| Aneurysm location (Acom, Pcom left, Pcom right, Other) |
| Free text describing aneurysm at other location |
| Ectatic vessel detected (Yes/No) |
| Location of ectatic vessel |
| Dissection (Yes/No) |
| Location of dissection (ICA left, ICA right, Vertebral artery left, Vertebral artery right, Other) |
| Free text describing dissection at other location |

## Supplemental Table 2C

| **Combined vascular and ischemic lesion parameters (categories)** | |
| --- | --- |
| MRA LAO related to lesion (Yes/No) |  |
| LAO related to lesion location (most proximal occlusion noted if multiple occlusions in ACA, MCA and PCA) | No occlusion visible |
|  | ACA - A1 segment |
|  | ACA - A2 segment |
|  | ACA - A3 segment |
|  | MCA - M1 segment |
|  | MCA - M2 segment |
|  | MCA - M3 segment |
|  | PCA - P1 segment |
|  | PCA - P2 segment |
|  | PCA - P3 segment |
|  | Basilar artery |
|  | Vertebral artery |
|  | Internal carotid artery (proximal) |
|  | Carotid T-occlusion (distal) |
|  | Multiple |
| Free text field describing LAO location | |

## Supplemental Table 2A-C

Clinical parameters and parameters of the structured MRI report used for the assessment within the MRI-Genie project. 2A) Ischemic lesion parameters, assessed on DWI, describing the variables associated with lesion location and number, and other characteristics including vascular territory. 2B) Vascular parameters, assessed on MRA, including the predefined vascular variables and the categories for each variable. 2C) Combined vascular and ischemic lesion parameters, assessed on DWI and MRA, used to determine LAO.

## Supplemental Table 3

| **Manufacturer** | **Number** | **Model** | **Number** |
| --- | --- | --- | --- |
| GE | 1002 | Signa | 975 |
| Philips | 957 | Achieva | 223 |
|  |  | Gyroscan | 223 |
|  |  | Eclipse | 137 |
|  |  | Infinion | 136 |
| Siemens | 804 | Symphony | 275 |
|  |  | Magnetom | 219 |
|  |  | Avanto | 151 |
|  |  | Sonata | 117 |
|  |  | Verio | 10 |
|  |  | Trio | 6 |
|  |  | Numeris | 1 |
| Marconi | 43 | Edge | 28 |
| Hitachi | 3 |  | |
| Toshiba | 1 |  |  |
| **Field Strength** | |  |  |
| 1.5T | 2010 |  |  |
| 3T | 340 |  |  |

## Supplemental Table 3

Distribution between manufacturers, scanner models and scanner field strengths for those examinations where the DICOM information included scanner information.
